# Enhanced Inhibitory Effect of Ultra-Fine Granules of Red Ginseng on LPS-induced Cytokine Expression in the Monocyte-Derived Macrophage THP-1 Cells

**DOI:** 10.3390/ijms9081379

**Published:** 2008-08-07

**Authors:** Hyoung-Cheol Lee, Radhakrishnan Vinodhkumar, Jang W. Yoon, Seong-Kyu Park, Chang-Won Lee, Hong-Yeoul Kim

**Affiliations:** 1 Lab of Biochemistry and Molecular Biology, Department of Biochemistry, College of Oriental Medicine, Kyung Hee University, #1 Hoeki-dong, Dongdaemoon-gu, Seoul 130–701, Republic of Korea; E-mails: dragoonlee@hanmail.net (H.C.L.); jwy706@khu.ac.kr (J.W.Y.); vininmail@gmail.com (R.V.); 2 HelixPharms Co., Ltd., Seoul, 130–701, Republic of Korea; 3 Oriental Medical Science Center, College of Oriental Medicine, Kyung Hee University, Seoul, Republic of Korea; 4 Department of Prescriptionology, College of Oriental Medicine, Kyung Hee University, Seoul, Republic of Korea; E-mail: comskp@khu.ac.kr (S.K.P.); 5 NT&BT Co., Ltd. 92-1, Haengsan-ri, Galsan-myeon, Hongseong-gun, Chungcheongnam-do, Republic of Korea; E-mail: ntbt@korea.com (C.W.L.)

**Keywords:** red ginseng, HAC-gearshift system, ultra-fine granules, anti-inflammatory effect, THP-1 cells

## Abstract

Red ginseng is one of the most popular traditional medicines in Korea because its soluble hot-water extract is known to be very effective on enhancing immunity as well as inhibiting inflammation. Recently, we developed a new technique, called the HAC-gearshift system, which can pulverize red ginseng into the ultra-fine granules ranging from 0.2 to 7.0 μm in size. In this study, the soluble hot-water extract of those ultra-fine granules of red ginseng (URG) was investigated and compared to that of the normal-sized granules of red ginseng (RG). The high pressure liquid chromatographic analyses of the soluble hot-water extracts of both URG and RG revealed that URG had about 2-fold higher amounts of the ginsenosides, the biologically active components in red ginseng, than RG did. Using quantitative RT-PCR, cytokine profiling against the *Escherichia coli* lipopolysaccharide (LPS) in the monocyte-derived macrophage THP-1 cells demonstrated that the URG-treated cells showed a significant reduction in cytokine expression than the RG-treated ones. Transcription expression of the LPS-induced cytokines such as TNF-α, IL-1β, IL-6, IL-8, IL-10, and TGF-β was significantly inhibited by URG compared to RG. These results suggest that some biologically active and soluble components in red ginseng can be more effectively extracted from URG than RG by standard hot-water extraction.

## 1. Introduction

Among the several species of the genus *Panax*, *Panax ginseng*, so called “Korean ginseng”, is the most popular ginseng in Korea. Several studies on *P. ginseng* suggest that it has many beneficial effects such as anti-inflammation, anti-oxidation, and anti-cancer activity [1]. A well defined component responsible for those effects in *P. ginseng* is known to be the ginsenosides (saponins) triterpene glycosides. Many different kinds of ginsenosides have been reported, but their amounts and composition can be varied depending on the types of *P. ginseng* such as undried, dried (white), or red ginseng [2]. Red ginseng has been used as a prophylactic medicine to enhance immunity as well as inhibit inflammation in various diseases for a long time in Korea. This type of ginseng is produced by a repeated process of steaming undried ginseng roots for 2–3 h and drying them.

By definition, cytokines are a large group of soluble extracellular proteins or glycoproteins which are functioning as intercellular signals and/or effectors. Based on the structural homologies of their receptors, they are classified into three family groups, namely interleukins (IL), interferons (IFN), and chemokines. In function, however, cytokines can be divided into the two groups; pro- or anti-inflammatory cytokines. The pro-inflammatory cytokines such as tumor necrosis factor-α (TNF-α), IL-1α/β, IL-12, and IFN-γ induce immunogenic and inflammatory responses [3–5], whereas the anti-inflammatory cytokines such as transformation growth factor-β (TGF-β), IL-4, IL-10, IL-11, and IL-13 inhibit such immunogenic and inflammatory responses. Therefore, fine balance between both pro- and anti-inflammatory cytokines is believed critical for host defense against the diseases. Supporting this notion, increasing evidence implies that the modulation of cytokine expression may provide an alternative therapeutic approach for various diseases [6, 7]. Such immunomodulation can be achieved by medicinal herbs because some of medicinal herbs were able to alter immune function through direct or indirect regulation by means of certain signaling molecules like cytokines [8, 9].

Recently, a new technique has been developed to make medicinal herbs into very fine particles with sizes ranging from 0.2 to 7.0 μm. This simple, but innovative pulverization technique has been named the HAC-gearshift system because the system involves a total of three consecutive pulverization steps; the first is in a hammer mill, the second in an air classifying mill, and the third in a classifying cyclone. The average size of the final particles through this system ranged from 0.2 to 7.0 μm. In contrast, the conventional pulverization technique produces relatively larger particles than the HAC-gearshift system does, with sizes averaging 127 μm.

In this study, we hypothesized that this physical modification from normal to ultra-fine granules of medicinal herbs by the HAC-gearshift system could enhance their medicinal effects. To test this hypothesis, the ultra-fine granules of red ginseng (URG) were formulated using the HAC-gearshift system and used to prepare the soluble extract by standard hot-water extraction. Finally, the soluble hot-water extract of URG was compared to that of the normal granules of red ginseng for their biochemical compositions and previously defined anti-inflammatory activities.

## 2. Results

### 2.1. Biophysical properties of URG prepared by the HAC-gearshift system

**Figure 1.**
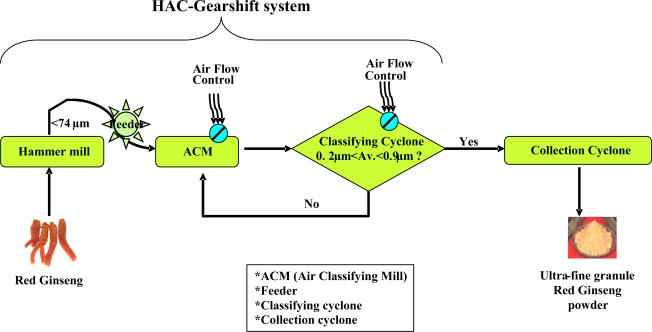
A schematic diagram showing the HAC-gearshift system.

**Figure 2.**
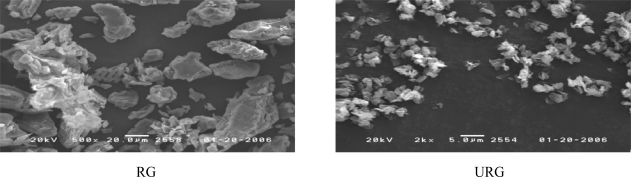
Transmission electron microscopy (TEM) of URG and RG. The average size of URG was approximately 3.5 μm (right panel), compared to RG (average 127 μm in size; left panel) which was pulverized by a conventional grinder.

Recently, we developed a new pulverization technique called the HAC-gearshift system, which allows producing very fine particles in sizes ranging from 0.2–7.0 μm (Figure 1). Using this simple system, red ginseng was processed as shown in Figure 1 and transformed into ultra-fine granules (also, see Experimental section). As expected, transmission electron microscopic analysis showed that the average size of URG was approximately 3.5 μm, while that of the normal granules prepared by the conventional pulverization system (RG) was about 127 μm (Figure 2).

**Figure 3.**
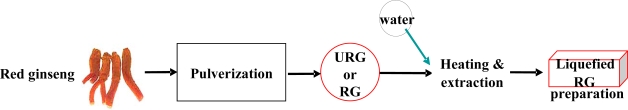
Procedure for hot-water extraction of URG and RG.

Therefore, we hypothesized that URG might be more efficient than RG for extracting some beneficial components of red ginseng because of their particle size differences. To test this, the soluble extracts from either URG or RG were prepared by standard hot-water extraction (Figure 3) and measured the amounts of the ginsenosides in those extracts using high-pressure liquid chromatography (HPLC; Table 1).

The results showed that the hot-water extract of URG contained about 2-fold higher amounts of the ginsenosides than that of RG. As a total, about 20.9 mg/g of the ginsenosides was extracted from URG, but only about 10.7 mg/g was extracted from RG. This indicates that URG can be more efficiently extracted by hot-water extraction than RG (Table 1).

### 2.2. Effects of LPS and phobol 12-myristate 13-acetate in TNF-α expression in the monocyte-driven macrophage THP-1 cells

Previous studies reported that the hot-water extract of RG had a significant anti-inflammatory effect and its biologically active components were the ginsenosides. Since the hot-water extract of URG contained significantly higher amounts of the ginsenosides than that of RG, it is possible that the URG extract may have higher anti-inflammatory activity than the RG extract. Therefore, we determined the abilities of URG and RG to induce the pro-inflammatory cytokines. To the end, any potential toxic effects of URG and RG were examined at first by measuring cell viability over the monocyte-driven macrophage THP-1 cells [11, 12]. Using the MTS assay, it was found that there was no toxic effect of URG and RG with increasing concentration up to 1,000 μg/mL (data not shown). Since cell viabilities to both URG and RG were slightly better at the concentration of 100 μg/mL than 1,000 μg/mL, 100 μg/mL of URG and RG have been used for the assays in this study (data not shown).

**Figure 4.**
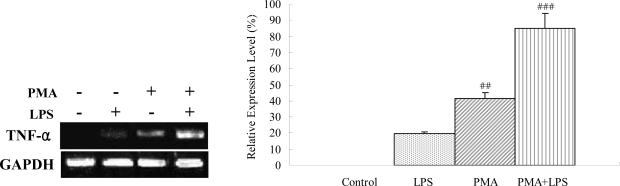
Effect of LPS (5 μg/mL) and 160 nM PMA on TNF-α expression in the THP-1 cells.

Since it is known that transcription expression of TNF-α is induced by LPS in the THP-1 cells unless the cells are differentiated by phobol 12-myristate 13-acetate (PMA), we examined transcription expression of TNF-α in the THP-1 cells with or without PMA, LPS, or both. Using quantitative RT-PCR, the results support the fact that TNF-α expression was not induced in the THP-1 cells without pre-treatment of PMA. The PMA-treated cells with LPS for 2 h could induce substantial expression of the TNF-α mRNA (Figure 4). In contrast, such induction was not observed in the cells untreated with PMA (Figure 4).

### 2.3. Effect of URG and RG on LPS-induced cytokine expression in the THP-1 cells

**Figure 5.**
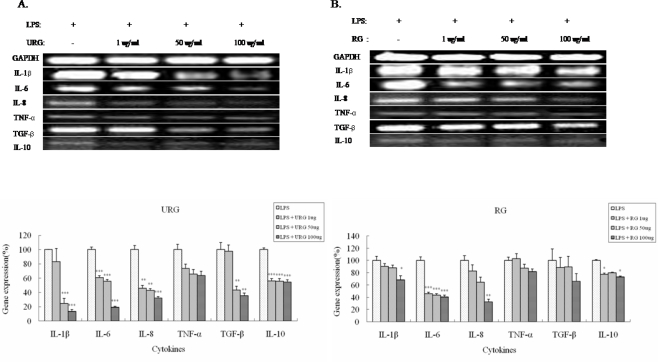
Cytokine profiling in the 160 nM PMA-treated THP-1 cells by URG (A) or RG (B).

In order to examine the effects of URG and RG on LPS-induced cytokine expression, transcription expression of the previously defined pro-inflammatory cytokines was examined by quantitative RT-PCR. The THP-1 cells were exposed to PMA for 72 h and then treated with LPS with or without URG, RG, or both as described above. As expected, both URG and RG extracts were able to down regulate expression of both pro- and anti-inflammatory cytokines including IL-1β, IL-6, IL-8, TNF-α, IL-10, and TGF-β (Figure 5). The observed inhibitory effects were dose dependent in both URG and RG extracts. However, the URG extract could inhibit expression of those cytokines more efficiently than the RG extract did. As the concentrations of URG and RG were increased to 1, 50, and 100 μg/mL, IL-1β transcription was decreased to 16%, 75%, 86% with URG and 9%, 11%, 31% with RG, compared to the control without either URG or RG. IL-6 transcription was decreased to 39%, 44%, 80% with URG and 54%, 56%, 59% with RG. IL-8 transcription was decreased to 54%, 57%, 68% with URG and 13%, 35%, 67% with RG. TNF-α transcription was also down regulated to 26%, 34%, 36% with URG and −3%, 12%, 18% with RG. Similarly, TGF-β transcription was also down regulated to 4%, 56%, 64% with URG and 11%, 10%, 34% with RG. Surprisingly, the anti-inflammatory effects of URG were very close to those of a well defined anti-inflammatory drug, PD98059, with an exception of TNF-α (Table 2). These results indicate much stronger anti-inflammatory activity of URG than RG.

## 3. Discussion

The ultra-fine granule is a basic feature of our HAC-gearshift system, whereby food or medicinal herbs are physically formulated as nano-particles by a broken cell wall technology. In order to evaluate and compare this system to the conventional pulverization system, red ginseng was applied to this system and compared to that prepared by the conventional pulverization system. When URG was compared with RG, we found that the soluble hot-water extracts of URG had more ginsenosides, the biologically active components of red ginseng, than those of RG as well as showed enhanced inhibitory effect on LPS-induced cytokine expression in THP-1 cells compared to those of RG.

Previously, it was well known that LPS, the endotoxin component of Gram-negative bacteria, is responsible for initiating the host responses causing inflammation [13]. This molecule is one of the most potent biological response modifiers so that the picomolar concentrations are sufficient to stimulate cells of the immune, inflammatory, and vascular systems [14]. A vast amount of information about the molecular mechanism of host defense responses and inflammatory mediators has been derived from studies using LPS as a stimulus [15]. Interestingly, LPS is not intrinsically toxic, but allows myeloid and/or non-myeloid cells to induce several genes encoding proteins associated with the hemodynamic and hematologic changes observed in inflammation [13, 16]. These inducible genes encode cytokines, adhesive proteins, and cellular enzymes which produce small pro-inflammatory mediators such as leukotrienes and prostaglandins (PGs). These substances are important regulators of both innate and adaptive immunity [17]. However, their uncontrolled expression can cause acute or chronic inflammatory diseases [17]. PD98059 is a well known LPS inhibitor and can selectively block the activity of MEK which subsequently inhibits both the phosphorylation and activation of MAP kinases *in vitro*. This inhibitor was discovered by screening a chemical library for inhibitors of the MAP kinase cascade [18]. Although the mechanisms behind the anti-inflammatory activity of red ginseng are unclear yet, we were surprised that the URG extracts showed very similar inhibition of pro-inflammatory cytokine expression in the LPS-stimulated THP-1 cells to PD98059, especially in expression of IL-1β, IL-6, and IL-8. Further study will be needed for defining a possible mechanism on the PD98059-like anti-inflammatory activity of red ginseng.

Although bacterial LPS is a well-defined inflammatory mediator, this molecule is also known as one of the best characterized monocytic mitogens, which, following interaction with its receptor CD14, induces first proinflammatory cytokines such as IL-1 and TNF-a and then anti-inflammatory cytokines such as IL-10. Regulation of pro- and anti-inflammatory cytokines has been suggested to be involved in distinct signaling pathways. For example, a recent study demonstrated IL-10 production in the LPS-activated THP-1 cells by transcriptional activation from the promoter sequence containing the consensus sequence for Sp1 and PD98059 failed to modulate IL-10 production [19]. Not surprisingly, our data showed that the LPS-stimulated THP-1 cells could induce both pro- and anti-inflammatory cytokines. However, it should be noted that red ginseng could inhibit both pro- and anti-inflammatory cytokine expression in the LPS-stimulated THP-1 cells. Given that the anti-inflammatory effect of red ginseng was well established, simultaneous repression of both pro- and anti-inflammatory cytokines by red ginseng seems not to be involved in causing severe inflammation. Instead, its repression of anti-inflammatory cytokines such as IL-10 may enhance antigen-driven activity of both Th1 and Th2 subsets. Supporting this notion, it has been reported that high levels of IL-10 were produced in patients with HIV infection [20]. It would be interested in identifying certain biological compounds in red ginseng that are responsible for altering transcription expression of both pro- and anti-inflammatory cytokines although the mechanisms behind that regulation are unclear yet.

Currently, corticosteroids are the most common pharmacological drugs to control inflammation in the clinic. But, these drugs have significant side effects, especially for long-term use. Therefore, there are serious interests in developing more efficient and safer anti-inflammatory drugs. By using LPS as a stimulus, our results demonstrate that the URG extracts showed a higher biological activity than the RG extracts. This could be explained by the fact that the soluble hot-water extract of URG contains the higher concentrations of the ginsenosides than that of RG, indicating that URG may allow the easier and more efficient extraction of some known or other as-not-yet characterized active components in red ginseng than RG. In previous studies, it is known that the major ingredients of ginseng roots are the ginsenosides, which have various physiological activities including anti-inflammatory activity, anti-allergic, endothelium-independent aorta relaxation, and anti-tumor effects [21–24]. In our study, however, the fold decreases in cytokine expression by URG was much greater than those expected from the biochemical analyses of URG and RG by HPLC. We can not explain this, but speculate that the biologically active components in red ginseng which are present in both URG and RG extracts may function synergistically on the THP-1 cells.

In conclusion, there seems a clear inhibitory effect of red ginseng on LPS-induced cytokine expression in cells because both URG and RG could inhibit expression of the major pro- and anti-inflammatory cytokines in the LPS-stimulated THP-1 cells. URG was more efficient than RG in this inhibitory effect because of the size differences: the average particle size of RG was about 127 μm whereas that of URG was about 3.5 um. Our demonstration of the higher inhibitory activity on LPS-induced cytokine expression of URG than RG suggests that the ginsenosides as well as other uncharacterized, biologically active components in red ginseng may be easily released out of the URG by standard hot-water extraction, compared to RG. Therefore, our HAC-gearshift system can be applied for other medicinal herbs because it provides an alternative and more efficient formulation of medicine.

## 4. Experimental Section

### 4.1. Preparation of URG by the HAC-gearshift system

RG was further pulverized into an ultra-fine granule URG using the HAC Gear Shift System (Figure 1). This system consists of three stages: in the first stage, RG was pulverized into fine powder of less than 74 μm by a hammer mill. In the second stage, it was fed into an ACM, which carries out the main pulverization process by high-speed turbulence air flow. The average size of particles could be controlled by the amount of air flow. In the final stage, the fine powder in ACM was fed into a classifying cyclone, which selected the particles of 0.2∼7.0 μm in size. If the particles were larger than 7 μm, those particles were returned to ACM. The proper sized particles were obtained from the collection cyclone chamber and the resultant nano-particles were defined as URG. The resultant URG and RG were mixed into the distilled water and the suspensions were further heated at 60° C for 2 h for extraction. The soluble hot-water extracts were used for the assays (Figure 3).

### 4.2. Reagents

URG and RG were prepared by decocting the dried herbs in boiling water (30 g/L) for 2 h as following: briefly, the extracts were freeze-dried for 48 h (Eyela, Japan), dissolved in the phosphate buffered saline (PBS), filtered using a 0.45μm-filter unit (Millipore, Ireland), and stored at 4° C until used. Before the assay, the dried extract was dissolved in distilled deionized water and vortexed for 2 minutes at room temperature [25]. These dried herbs were obtained from NT&BT (Korea). PMA (phorbol 12-myristate 13-acetate), LPS (lipopolysaccharide from Escherichia coli), PD 98059 [26] were purchased from Sigma-Aldrich (U.S.A).

### 4.3. Transmission electron microscopy (TEM, JEOL, Japan)

URG and RG were embedded into pure embedding media in the beam capsules. The embedded blocks were kept at 50° C for 12–14 hours. The temperature was raised to 60° C and kept for 24–48 hours. The blocks were trimmed and thick sections (0.5 to 2.0 m) were cut to observe under an optical microscope. The area to be examined under TEM was selected and the blocks were further trimmed. The ultra thin sections (2 μm) were lifted onto a carbon grid and double-stained with uranyl acetate and lead acetate. The grids were placed in a watch glass containing a small amount of uranyl acetate and lead citrate. The grids were placed in a watch glass containing small amount of uranyl acetate and stained for 10–15 minutes. The grids were washed in two lots of 50% ethanol and two lots of double distilled water with continuous agitation. After drying on the filter paper, the grids were placed in a watch glass containing a few mL of lead citrate and stained for 5 to 10 minutes. The grids were washed briefly in 0.02 M sodium hydroxide and then in two lots of distilled water. After drying the grids, they were applied for TEM analysis.

### 4.4. HPLC analysis

The soluble hot-water extracts of URG and RG were further analyzed at NT&BT (Korea) for their biochemical compositions by HPLC equipped with a C18 column and an auto-sampler Waters 717 plus.

### 4.5. Cell culture conditions

The THP-1 cells, known as the human monocytic leukemia cells, were obtained from Korean Cell Line Bank (Seoul, Korea). The cells were prepared and resuspended in RPMI 1640 medium (Gibco, U.S.A.) supplemented with 10% (v/v) heat-inactivated fetal bovine serum (FBS, Gibco) and 1% antibacterial antifungal solution (Gibco). They were maintained in a humidified incubator (Sanyo, Japan) at 37° C in presence of 5% CO_2_.

### 4.6. PMA treatment

160 nM PMA (Sigma) in RPMI medium was added to differentiate the THP-1 cells for 72 h at 37°C for all the cytokine assays through this study. The criteria for the differentiation of the THP-1 cells, adherence, morphological changes, and changes in expression of the cell surface makers such as integrin, FcγRI, CD4, and MHC class II antigen were applied. The PMA solution was prepared by dissolving PMA in sterile dimethylsulfoxide (DMSO) (Sigma). The stock solution was stored frozen at −20° C. Immediately prior to use, the PMA stock solution was diluted in RPMI medium to 160 nM at the final concentration.

### 4.7. Cell viability

Cell viability was determined by using the 3-(4,5-dimethylthiazol-2-yl)-5(3-carboxymeth oxyphenyl)-2-(4-sulfophenyl)-2H-tetrazolium (MTS) kit (Promega, U.S.A.) according to the manufacture's directions [27]. For analysis of cell toxicity by URG and RG, cells were pre-treated with PMA for 72 h, followed by treatment of URG or RG at various concentrations (0 mg/mL to 1 mg/mL) for 2 h. The control group was treated with the same amount of PBS. The MTS labeling reagent (20 μL) was added to each group and incubated for 2 h at 37° C. The absorbance of each well was measured at 490 nm using the Soft Max ELISA reader (Molecular Devices, U.S.A.). The optical density (OD) was calculated as the difference between the reference wavelength and the test wavelength as following: The percent of cell viability = [A490/nm of drug treated cells/A490/nm of control cells] × 100.

### 4.8. Macrophage differentiation and stimulation

The THP-1 cells were seeded onto an φ 100mm^2^ (1 × 10^6^ cells) for experiments. The cells were pre-treated with 160 nM of PMA for 72 h and subsequently stimulated with 5 μg/mL of *E. coli* LPS with or without URG, RG, or both for 2 h in a humidified incubator (Sanyo, Japan) at 37° C in presence of 5% CO_2_.

### 4.9. Reverse transcription (RT)-PCR analysis

Total cellular RNAs were isolated from the THP-1 cells treated with Trizol (Gibco). The cDNA was synthesized from 1 μg of total RNA using 200 U Moloney Murine Leukemia Virus (M-MLV) reverse transcriptase (Invitrogen) with a total reaction volume of 20 μL. The resultant cDNA was amplified using the gene-specific primers (Table 3). The amplified PCR products were analyzed using an Image Documentation System (GelDoc 2000; Bio-Rad) with the Quantity One software (Bio-Rad). The 1-kb DNA size marker (Fermentas, Canada) was run in parallel to estimate the size of the amplicon.

### 4.10. Statistical analysis

All the results were presented as the means ± SEM. One-way ANOVA with Tukey’s HSD test was used to determine their significance. Statistical analysis was carried out using the GraphPad Prism program (Prism 4.0 for Windows). The significance was defined with the following symbols: * P<0.05, ** P<0.01, *** P<0.001 against to LPS treatment sample and #<0.05, ##<0.01, ###<0.001 against to control.

## Figures and Tables

**Table 1.** Quantitation of the ginsenosides in the hot-water extracts from URG and RG (1 gram each) by HPLC.

Ginsenosides^A^		RG (mg/mL)	URG (mg/mL)	Fold increase (URG vs. RG)
PD	Rb1	2.32	5.00	2.16
	Rb2	1.05	2.41	2.29
	Rc	1.10	2.50	2.27
	Rd	0.63	1.70	2.69
	Rg3s	0.84	1.70	2.02
	Rg3r	0.39	0.73	1.87
	Rh2s	0.14	0.28	2.00
PT	Re	1.01	2.17	2.14
	Rf	0.58	0.87	1.50
	Rg1	0.60	0.95	1.57
	Rg2s	0.83	1.49	1.79
	Rg2r	0.87	0.59	0.68
	Rh1	0.33	0.48	1.45
Total sum		10.69	20.87	1.95

^A^Ginsenosides are characterized according to the number and position of sugar moieties on the sterol chemical backbone structure. Based on their structural differences, ginsenosides are divided into three main categories, the 20(*S*)-protopanaxadiol (PD), 20(*S*)-protopanaxatriol (PT), and oleanane families. The PT family chemically differs from the PD family by the addition of one hydroxyl group at C-6. Gisenosides Rb1, Rb2, Rc, Rd, Rg3s, Rg3r, and Rh2s belong to the PD family whereas ginsenosides Re, Rf, Rg1, Rg2s, Rg2r, and Rh1 belong to the PT family [10].

**Table 2:** Transcription expression of the pro-inflammatory cytokines in the PMA-treated THP-1 cells by URG (100 ug/ml), RG (100 ug/ml), or 10 nM PD98059.

Cytokines^A^	Control	LPS	LPS+URG	LPS+RG	LPS+PD98059^B^
IL-1β	0.0	100 ± 0.2	13.7 ± 2.4	59.2 ± 6.7	4.9 ± 1.3
IL-6	0.0	100 ± 3.6	19.7 ± 0.8	60.5 ± 2.8	13.2 ± 0.5
IL-8	0.3	100 ± 5.5	32.3 ± 1.8	48.6 ± 4.1	30.5 ± 1.7
TNF-α	6.5 ± 0.5	100 ± 7.4	66.0 ± 5.9	94.0 ± 4.2	11.8 ± 1.0
TGF-β	0.7 ± 0.3	100 ± 9.6	35.6 ± 3.5	86.5 ± 12.5	33.4 ± 3.1

^A^ A well known LPS inhibitory drug

^B^ Transcription expression was expressed in the mean ± standard error

**Table 3:** Oligonucleotide sequences in this study

Name		Oligonucleotide sequence (5′–3′)	Expected PCR product (bp)
GAPDH	sense	GGTGAAGGTCGGAGTCAACGG	500[28]
	anti-sense	GGTCATGAGTCCTTCCACGAT	
IL-1β	sense	GGGCCTCAAGGAAAAGAATC	470 [29]
	anti-sense	AGCTGACTGTCCTGGCTGAT	
IL-6	sense	AAAGAGGCACTGGCAGAAAA	408 [29]
	anti-sense	GAGGTGCCCATGCTACATTT	
IL-8	sense	AGGGTTGCCAGATGCAATAC	378 [30]
	anti-sense	AGACTAGGGTTGCCAGA	
TNF-α	sense	AGCCCATGTTGTAGCAAACC	424 [31]
	anti-sense	CCAAAGTAGACCTGCCCAGA	
TGF-β	sense	GACTGCGGATCTCTGTGTCA	480
	anti-sense	CTGGTCTCAAATGCCTGGAT	
